# Semi-Supervised Segmentation Framework Based on Spot-Divergence Supervoxelization of Multi-Sensor Fusion Data for Autonomous Forest Machine Applications

**DOI:** 10.3390/s18093061

**Published:** 2018-09-12

**Authors:** Jian-lei Kong, Zhen-ni Wang, Xue-bo Jin, Xiao-yi Wang, Ting-li Su, Jian-li Wang

**Affiliations:** 1 School of Computer and Information Engineering, Beijing Technology and Business University, Beijing 100048, China; kongjianlei@btbu.edu.cn (J.-l.K.); wzhenni@163.com (Z.-n.W.); jinxuebo@btbu.edu.cn (X.-b.J.); sutingli@btbu.edu.cn (T.-l.S.); wangjianli@btbu.edu.cn (J.-l.W.); 2 Beijing Key Laboratory of Big Data Technology for Food Safety, Beijing Technology and Business University, Beijing 100048, China

**Keywords:** multi-sensor joint calibration, high-dimensional fusion data (HFD), supervoxel, Gaussian density peak clustering, sematic segmentation

## Abstract

In this paper, a novel semi-supervised segmentation framework based on a spot-divergence supervoxelization of multi-sensor fusion data is proposed for autonomous forest machine (AFMs) applications in complex environments. Given the multi-sensor measuring system, our framework addresses three successive steps: firstly, the relationship of multi-sensor coordinates is jointly calibrated to form higher-dimensional fusion data. Then, spot-divergence supervoxels representing the size-change property are given to produce feature vectors covering comprehensive information of multi-sensors at a time. Finally, the Gaussian density peak clustering is proposed to segment supervoxels into sematic objects in the semi-supervised way, which non-requires parameters preset in manual. It is demonstrated that the proposed framework achieves a balancing act both for supervoxel generation and sematic segmentation. Comparative experiments show that the well performance of segmenting various objects in terms of segmentation accuracy (F-score up to 95.6%) and operation time, which would improve intelligent capability of AFMs.

## 1. Introduction

For the foreseeable future, autonomous forest machines (AFMs) will play a central role in harvesting, tending and forest management because lots of artificial and natural forests are facing with poor forest quality on a large-scale [1]. Currently, the proportion of good quality forest resources is very small, which directly affects the economic potential and ecological value of the whole forestry industry. Scientific tending and harvesting operations, which rationally adjust the mixed structure and wood quality of forest, can improve the level of forestry production in a relatively short time [2]. However, traditional artificial technology relying on the high-cost and low-efficiency labor could not meet the urgent quality improvement of modern forestry. Therefore, replacing human beings with AFMs rapidly to engage in dangerous and heavy works including tending and harvesting have been a core focus, which will increase the efficiency and value of unit labor in handing with quality promotion of large-scale forest lands. Nevertheless, the automatic or semiautonomous ways presented that can work without operators cannot perceive the complex forest environments accurately as drivers, because so many complex factors need to be considered in the maneuvering of forest machines in harvesting operations that full automation would be extremely difficult. Many problems still remain to be addressed before the field of AFMs can be widely expanded including: which trees should be harvested or tended, where the harvester should be positioned and what suitable driving routes should be taken? To this end, an efficient environment-aware system that could facilitate decision-making in those complex work described above should be constructed before a forest machine can fully and authentically automate the harvesting and tending process, which will relieve fatigue and stress of drivers and improve overall productivity and efficiency of forestry workload [3]. 

In comparison to the structured environment in agriculture or the semi-structured outdoor environment in urban traffic, the forest environment is much more challenging for operation and perception of AFMs since the various objects of interest are surrounded by massive areas of dense shrubs, dead trees and fallen objects. In addition, forest trails are rarely straight or flat, and obstacles are common, the AFM itself has more problems in the process of moving due to high amount of logging residues on the ground surface and sometimes high variability of the soil-bearing capacity [4]. In order to improve the capacity of environmental perception and complex decisions in forest operations, most of the current AFMs combine several sensors to compensate for the drawbacks of each sensor and to merge various information into a single percept about the nearby environment [5]. Although multi-sensor fusion technology has become the standard techniques for AFMs to identify objects, select roads and decide to execute the best operation, separating individual objects and backgrounds from forest environments is extremely demanding and raises problems that have not yet been satisfactorily resolved, which becomes the main challenge in forestry autonomous awareness and navigation systems [6].

Many previous works dealing with this perception issue did so by solving a semantic segmentation problem, aimed at determining which objects of the input data correspond to harvesting and tending operation, and detecting which areas and trails are suitable for driving. Segmentation of images and point clouds is an important capability for AFMs in unstructured forestry scene, which is a prerequisite for solving subsequent tasks such as navigation or reconstruction. The basic process of segmentation is labeling each measurement unit, so that the pixels or points belonging to the same surface or region are given the same label [7]. However, processing unstructured and massive data (including laser point clouds, visible images, thermal infrared photos etc.) obtained by different sensors is a much harder problem. 

On one hand, most of segmentation methods are proposed to handle with a single type of sensor data (images or point clouds), which does not take full advantage of higher-dimensional fusion data (HFD) captured by the multi-sensor measuring system of AFMs. Additionally, those algorithms are focusing on point-level data or patch-level supervoxel with a fixed size, which is not suitable for the size change of HFD caused by the occurrence of spot-divergence in complex forestry scenes. Thus, finding approaches that can directly operate on size-changed HFD in an effective and affordable way is still largely open in application requirements of AFMs. On the other hand, the existing segmentation works falling into the cluster category in Euclidean space are supervised-based methods, which encourages users to try many different input thresholds, therefore, increases the chance of selecting good input values for better results. Consequently, such a process usually requires numerous parameters of human intervention and can be quite time consuming. This strategy may perform well in simple and sparse datum obtained from the city vehicles or indoor robots in structured environment. However, it is difficult to deal with the noise-filled HFD of forest environment in real-time, which even lead to inaccurate results of segmentation.

Aiming to improve the segmentation performance of the multi-sensor measuring system for AFMs, we propose a semi-supervised segmentation framework based on a size-changed supervoxel, which takes the spot divergence of each HFD into account and produces the valid feature vector covering the spatial, visual and thermal information at a time. On the basis of precomputed supervoxelization, we further extend the traditional density peak clustering method in Gaussian constraint to solve the sematic segment problem of different objects in complex scenes. This framework has two major benefits: (a) it provides a patch-level process that every supervoxel can describe the variational size of HFD; (b) sematic objects can be segmented without the artificially preset of clustering central number or convergence thresholds, which gives an opportunity to promote the segment performance in the term of accuracy and operation time. 

The rest of this paper is organized as follows: some related works are introduced in Section 2. Section 3 briefly presents our multi-sensor measuring system of AFMs. Section 4 presents the principle and notations of our segmentation framework. Experiments are conducted in Section 5. Finally, we conclude our work in Section 6.

## 2. Related Works

Focusing on HFD, there is a growing tendency of innovative methods for the treatment and analysis of these data, aimed ultimately to exploit in-depth the informative value of semantic segmentation. The early attempt to group segmentation methods followed the works of spatial transformation by converting 3D/2.5D point clouds into 2D depth images, which could be deposed with proven image segmentation techniques [8]. However, those methods lacked the geospatial information of 3D point clouds. Consequently, many further research paid attention on 3D-based segmentation methods able to understand a complex scene directly [9]. Those algorithms fell into the basic combination of the original point-level data and model-fitting method, which did take visual information and reflectivity intensity of HFD in account. In order to promote the segment performance processed in a point-wise manner, the following works of extracting supervoxels for 3D point clouds began to take multi-sensor information fusion and machine learning methods into account [10,11]. In this section, we will review some representative algorithms that are related to sematic segmentation of HFD.

### 2.1. Point Clouds-Based Segmentation Method

The traditional segmentation methods are dividing large amount of unstructured 3D point clouds into a certain number of independent objects with special semantics according to spatial distribution characteristics. Over the past decade, several algorithms for object extraction from 3D point clouds have been reported by researchers. Euclidean clustering segmentation was based on defining a neighbourhood of radius and all the points within the sphere of radius are belong to one cluster [12]. Although such methods allow a fast segmentation, they may produce inaccurate results in case of noise and uneven density of point clouds, which commonly occur in point clouds.

For higher accuracy, model-fitting methods were proposed with the observation that many objects could be decomposed into geometric primitives like planes, cylinders and spheres [13]. For example, the cylinder was usually fitted onto point clouds of forest scenes to distinguish the trunks which were conform to the mathematical representation of the primitive shape. As part of the model fitting-based category, two widely employed algorithms were the Hough Transform (HT) and the Random Sample Consensus (RANSAC) approach. Compared to the HT only detects fixed shapes, the RANSAC method was used to extract shapes by randomly drawing minimal data points to construct candidate shape primitives, which were checked against all points of dataset to determine the appropriate value. The model-fitting method has been adapted to segment tree stems in forestry scene. Ref. [14] proposed hierarchical minimum cut method based on that the detected trunk points are recognized according to pole-like shape. By detecting the repetitive appearance of cylindrical segment units, this method isolated individual trees from point clouds of forest scene and achieved good balance in terms of accuracy and correctness. However, the segmentation quality of the model fitting-based algorithms is sensitive to the point clouds characteristics (density, positional accuracy, and noise) and is over-reliance on predictive shapes and parameters, which lack of adaptability to segment for various objects in forest scenes.

An alternative was the region growing approach involving two stages: identification of the seed points based on the curvature of each point, and growing them based on predefined criteria such as proximity of points and planarity of surfaces. This method and several variations were presented for 3D point clouds segmentation. For example, [15] performed a marker-controlled region growing segmentation using a treetop as the seed surface and the angle and height between the neighboring triangles for the growing. However, the region growing methods strongly depends on multiple criteria, such as the location of initial seed regions and curvatures of points near region boundaries. Moreover, the high point density requires a large amount of computer resources for spatial searching if the original LiDAR points are processed directly in those methods. Thus, octree construction providing an efficient spatial index with high position accuracy was combined with the region growing methods to detect planar segments, which realized better point cloud management and provided faster refinement process [16]. Similarly, [17] proposed an initial-to-fine algorithm performed on an octree-based representation of the input point cloud to extract stem-based initial segments. Then the output was then passed through a refinement segmentation of overlapped canopy, which can reduce technical difficulties and effectively separate neighboring trees even if their canopies are overlapped. In these works, the partition of a point cloud was achieved by an octree structure. Local patches were then extracted according to the leaves of the octree. The number of local patches is related to the number of whole points and the size of octree leaves. A major limitation of this method is that the interior shape structure is discarded. Further, those methods are not particularly robust as has been shown experimentally in part because the segmentation quality strongly depends both on multiple criteria and the selection of seed points/regions. 

As demonstrated above, point clouds-based algorithms are well established as robust methods for segmenting dense 3D point clouds in acquired in urban areas. However, these works have some disadvantages in dealing with large 3D data sets or scenes with complex geometries. As these algorithms only use all individual points, the computational cost and significant processing time are very high, making it impractical for real time applications. Besides, the raw point clouds from terrestrial laser scanner (TLS) or mobile laser scanner (MLS) often exhibit unorganized stripe structures due to the rotary scanning mechanism. These structures make the point clouds difficult in providing any information on local surfaces, which help extracting the inside/outside of the underlying feature for efficient segmentation. Since point clouds are unstructured and often massive, it is sought to reduce these points by grouping together or removing redundant or un-useful points for improving the segmentation quality. 

### 2.2. Supervoxel Process

In order to accelerate the existing segmentation with 3D point clouds processed directly in a point-wise manner, the patch-level methods have been proposed by clustering the individual 3D points together to form over-segmented voxels. In order to create the voxels, a 3D point is selected as center and all 3D points in the vicinity are selected with a fixed diameter (equal to maximum voxel size) to determine an actual voxel. With the voxel representation, the point clouds can first be divided into a number of patches and the processing can then be operated in a patch-wise manner. After the voxelized process, the 3D model can not only maintain the surface shape of the object, but also effectively describe the internal distribution. Since the number of patches is much smaller than the number of points in a point cloud, the efficiency of point clouds processing can be significantly improved. In the work of [18], volumetric 3D model was proposed to explicitly representing the forestry scenes, with the details of the trees and the surrounding unknown areas represented accurately. However, the voxelization of point clouds lacks of the fusion information including color, texture, thermal and reflectivity obtained by other sensors. This make the local description capabilities of each voxel degraded and limit the segmentation performance in complex applications of forest environment.

To gaining the higher representation than the voxel, some patch-wise segmentation applied supervoxels as basic elements to cope with HFD of complex tasks. These methods are inspired by the superpixel approaches that have been widely used in image analysis and processing. A typically superpixel method was the SLIC (Simple Linear Iterative Clustering) algorithm based on gradient-ascent theory in which the relationship between the color similarity and the spatial distance was used to form the cluster centers of superpixels [19]. Another representative method was the Superpixels Extracted via Energy-Driven Sampling (SEEDS) algorithm based on graph-based theory, which started with the color distribution and discriminates edge shape of each superpixel iteratively to achieve superpixel partitioning [20]. 

Currently, many supervoxel-based segmentation methods are the simply extension of 2D superpixel segmentation to the domain of 3D volumes. Reference [21] proposed a Voxel Cloud Connectivity Segmentation (VCCS) method to take full advantage of 3D geometry information. In this work, the points with similar normals, colors, and Fast Point Feature Histograms (FPFHs) were clustered into a supervoxel. Similarly, the proposed SEED-3D algorithm was designed to minimise the cost of the shortest path in the weighted graph with consideration of characteristics of the sensor for complex urban environment. The performance showed the better boundary recall and under-segmentation result [22]. These kinds of supervoxel methods seem to not only be suitable for real 3D volumetric data, but also be appropriate for video with object occlusion and moving objects. Similarly, [23] proposed a novel voxel-related Gaussian mixture model for supervoxel segmentation to address the problem of new and moving objects in continuous frames. According to the experiments, the proposed method performed well in terms of segmentation accuracy while possessing a competitive computing. In particular, the supervoxels have showed as the best processing unit for the individual tree segmentation from LiDAR point clouds in urban environments. Reference [24] proposed an automatic method for the individual tree segmentation (ATS) based supervoxel generation. With the preprocessing of extracting tree points, the supervoxel was defined as a polyhedral region consisting of homogeneous points. Then assigning other points to optimize centers obtained complete supervoxels and delineate trees from complex scenes. This method overcame two main drawbacks in the commonly used tree point assignment strategy, including the low efficiency caused by assigning the index to each point and the assignment of different tree indexes for homogeneous points.

### 2.3. Supervoxel-Based Segmentation Method

As describing the local characteristics of point clouds effectively and reducing the processing time of segmentation, the supervoxels are selected as the basic processing unit for patch extraction of HFD in this paper. When the 3D point clouds or HFD are converted into some supervoxels, the next issue is to group these patches to segment into distinct objects. Usually for such task, [25] proposed a link-chain method instead to group these s-voxels together into segmented objects. However, this method has many features and parameters which need to be adjusted manually in order to obtain better results with very long computational time. Therefore, segmentation algorithms based on K-means clustering were applied to group set of supervoxels into different objects using few attributes/features. In [26], the feature distances between cluster centers and the neighborhood supervoxels are minimized to segment street trees from 3D point clouds. Since the choice of neighborhood strongly influences segmentation results of the K-means clustering methods, it is difficult to segment the boundary supervoxels with abundant features. Thus, a refinement phase was necessary to test whether the supervoxel was within the same cluster. The extracted segmentation based on hierarchical clustering was proposed to compute geometrical and radiometric characteristics (position, surface normals, reflectance etc.) of each supervoxel for forestry scene segmentation [27]. Similarly, a novel Density Based Spatial Clustering of Applications with Noise (DBSCAN) clustering algorithm was presented to cluster any dimensional data including terrestrial point clouds and HFD [28]. 

As described previously, those clustering segmentation methods require artificially determining the number of cluster centers or selecting convergence thresholds, which lacks automatic adaptability. Furthermore, these methods are all supervised model as they rely on a set of provided training examples (features) to learn how to correctly perform a task. While high-quality features can enhance algorithm performance, and can also causing computationally expensive in large datasets [29]. Hence, the partially un-supervised extraction of scene structures from 3D point clouds or HFD has been found to be an attractive approach to urban and forestry scene analysis, because it can tremendously reduce the resources and time consumption of data analyzing for subsequent tasks and other applications of AFM in forest environments. Consequently, the density peak clustering (DPC) algorithm is adopted to construct our proposed segmentation method on the basis of the idea that cluster centers are characterized by a higher density than their neighbors and by a relatively large distance from data with higher densities. The DPC has been widely applied to the problem of classification as the clusters are recognized automatically regardless of their shape and of the dimensionality of the space [30]. However, it cannot be directly applied to supervoxel segmentation because its accuracy excessively depended on the suitable threshold estimated on the basis of empirical experience [31]. What’s worse, it does not encode the constraint on dimensional consistency of feature vector for each supervoxel. Thus, a new Gaussian way is proposed to automatically extract the optimal value of threshold by using the normalized feature distance. For any multi-sensor fusion data of forestry scenes to be clustered, our proposed method can extract sematic objects with semi-supervised way from the supervoxels dataset objectively instead of empirical estimation. The details of the algorithm process are explained in the following sections.

## 3. Multi-Sensor Measuring System

**Figure 1:**
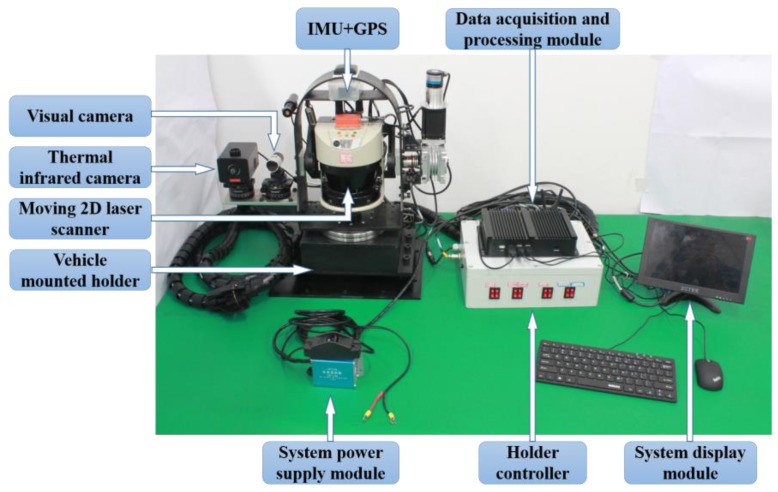
The AFM multi-sensor measuring system.

According to the environmental characteristics of AFMs’ operation, the vehicle-mounted holder is designed to carry a moving 2D laser scanner, thermal infrared camera and visual camera to build the real-time measuring and perception systems as shown in Figure 1. The camera could obtain real-time visible light information in the forest environment. This sensor has a wide-angle view field with 75° × 75° and a focal length with 0.1 m to 10 m, which has produced images with the resolution of 1920 × 1080 pixels at 20 frames/second speed. An ARTCAM-320-THERMO (ARTRAY CO., LTD, Tokyo, Japan) is selected as the thermal infrared device. Its measurement temperature range is from −40 to 150 °C. We set image resolution as 480 × 640 and the speed rate as 20 frames/second to detect the forestry objects. Finally, the LMS511-20100 PRO type laser scanner produced by SICK Corporation (Waldkirch, Germany) is used as a non-contact scanner. Its wavelength is 905 nm, which is safe and reliable for the human eye. To acquire abundant tree features with adequate resolution from the laser scanning measurements taken in the forest, the scanning angular resolution is set to its minimum value 0.1667°. Then the scanning angle is set to −5° to 185° and maximum scanning distance is 50 m. The measurement points corresponding to the surrounding contour is output in hexadecimal format to form the raw point clouds via the Ethernet interface at the frequency of 100 Hz. In this study, we extended the 2D scanning model to 3D scanning model combining the pan/tilt motion of the vehicle-mounted holder with the internal motor motion of 2D laser device. Therefore, the horizontal direction parameter of 3D points depended on the setting result of the laser scanner above, and the vertical direction parameter was determined by the vehicle-mounted holder. Here, we set the scanning angular resolution as 0.1°. Similarly, the scanning angle is set to −70° to 70° (0° is parallel to the ground) and the scanning frequency is set to 10 Hz. 

**Figure 2:**
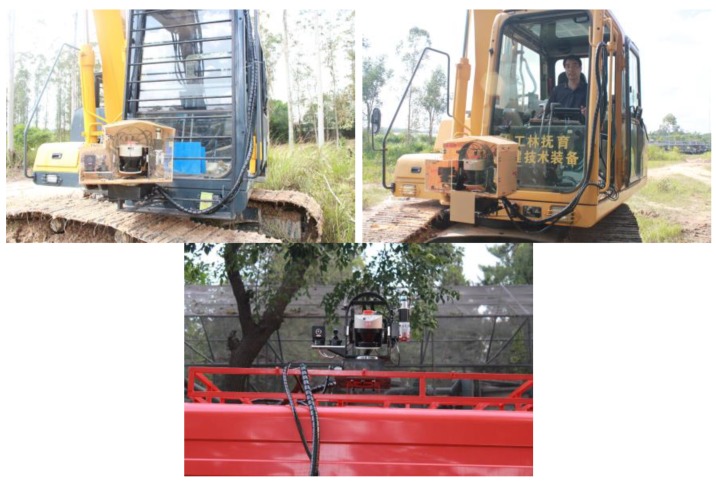
Measuring system installed at different forestry machines for operations experiment.

Through multi-sensor cooperation, information such as distance, position distribution, color and surface temperature of objects in the forest area can be directly obtained and stored through the host control software in the data acquisition and processing module. This software also was used to control the working states of all sensors, vehicle-mounted holder and the system display module. The whole system was equipped on different forest machines (including forestry firefighting vehicles, forestry cutting and harvesting equipment, forestry tending and breeding equipment as shown in Figure 2) with proof level of IP67, which can effectively prevent the entry of rain or dust and be adapted to the damage conditions in the actual forestry areas. 

The multi-sensor data acquisition experiment of forest scenes was conducted in southern and northern forest farms in different seasons. The southern experiment was conducted in the artificial eucalyptus area of Qinzhou (Guangxi Province, China) where the diameter at breast height (DBH) was more than 16 cm and the height was more than 15 m. The experiment was carried out from July to November with the high temperature above 35–42 °C. In order to further enrich the measurement objects and scenes, we also selected the Jiufeng forest farm in Beijing for experimentation with various tree species including larch, fir, birch, etc. The experiments were carried in typical cold northern weather conditions with temperatures from −21 °C and 9 °C. 3D point clouds, visible images, and thermal infrared images of various objects and obstacles under different scenarios were acquired to form the multi-modal database of the forest environment. Based on the multi-sensor technology foundation, we focused on practical issues for AFM application in complex environments and carried out the sematic segmentation framework including subsequent four steps: multi-sensor joint calibration, spot-divergence supervoxelization, feature vector extraction and Gaussian density peak clustering as described in the following sections.

## 4. Methodology

### 4.1. Multi-Sensor Joint Calibration 

**Figure 3:**
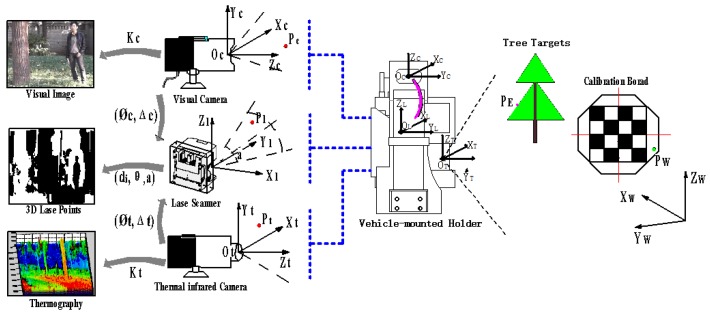
The joint calibration schematic of the thermal infrared camera, visible camera and 3D laser scanner for HFD.

Each sensors’ data in the multi-model database has its own characteristics. Compared with the visible and thermal infrared images, the 3D point clouds have a larger field of view, but the density of point clouds is relatively sparse and noisy compared to images. To integrate the advantages of different sensors for segmentation, it is necessary to perform multi-sensor data preprocessing and fusion on the basis of analyzing the various sensors’ conditions. According to the working principle of laser scanners, the measuring value of a laser beam is influenced by the reflectance of objects and the returned energy of the laser beams, which makes laser point clouds filled with discrete and systematic noise. Therefore, this paper uses a Gaussian weighted filter for removing discrete points in the laser scanner data which have the farther distance from all nearby point clouds. Then the average smoothing filter is following to suppress the systematic noise in the point clouds, making the surface of the object much smooth. Based on the noise processing, this paper conducts a joint calibration study of multiple sensors. Its purpose is to realize the original data conversion relationship between various sensor coordinate systems and achieve pixel-level data fusion. The joint calibration schematic is shown in Figure 3.

The entire multi-sensor joint calibration process consisted of two stages: internal calibration of each sensor and external calibration between them. In the internal calibration stage, we applied the linear pinhole imaging model on the basis of the nonlinear distortion to descript the internal geometric projection process in the visible and thermal infrared cameras. We took an octagonal calibration plate with checkerboard to calculate the internal parameters including focal length, distortion etc. By using the Zhang calibration method of [33], the visible camera’s coordinate $X^{cam}=\;{\left[X_{c},Y_{c},Z_{c}\right]}^{T}$ and the thermal infrared camera’s coordinate $Y^{temp}\;=\;{\left[X_{t},Y_{t},Z_{t}\right]}^{T}$ projected by the corresponding point in the world coordinate were confirmed to construct the joint calibration model. Meanwhile, the internal coordinate relationship of 3D point clouds by combining the pan/tilt motion of the vehicle-mounted holder with the 2D laser device needs to be analyzed. Since the center of the 2D laser scanner $O_{l}$ and the pan/tilt center $O_{L}$ did not coincide, which leaded the laser scanning plane rotated around $O_{L}$ with a certain distance d and angle δ. We defined the initial position of the 3D laser device when the plane of the $O_{l}$ center is parallel to the ground. Then the internal calibration expression of 3D laser device is modeled as:

(1) $$J^{lase}=\;\left[\begin{array}{c} X_{L}\\ Y_{L}\\ Z_{L} \end{array}\right]\;=\;\left[\begin{array}{c} D\cos(n_{1}\eta +\theta_{0})•\sin(n_{2}\varepsilon +\delta )]\\ \left[D\sin(n_{1}\eta +\theta_{0})-d_{x}]•\sin(n_{2}\varepsilon +\delta \right)\\ \left(H+d_{y})•\cos(n_{2}\varepsilon +\delta \right) \end{array}\right]\;$$

where $d_{x}$ and $d_{y}$ are the horizontal and vertical offset of d, D is the distance value measured by the point cloud, and $\theta =n_{1}\eta +\theta_{0}$ is the horizontal distribution angle of the laser beam formed by the horizontal angular resolution η and the horizontal laser beam sequence $n_{1}$, $\theta_{0}$ represents the initial angle of the working range of the 2D laser scanner. $n_{2}\varepsilon$ is formed by the tilt angle resolution ε and the sequence of scan plane layers $n_{2}$. According to the parameters of the laser scanner and the vehicle-mounted holder preset in Section 3, we acquired the quantitates η=0.1667, ε=0.1, $\theta_{0}=-5$, H=1.6. Applied the method in [33], we used the outer edge characteristics of the calibration plate consisted of the points $\left(D,n_{1},n_{2}\right)$ to confirm the internal parameters $d_{x}$, $d_{y}$, δ in 3D laser scanner coordinate. With the internal parameters of three sensors determined, we established fusion coordinate system based on the 3D laser scanner, and built a joint calibration parameter solving model between multiple sensors:

(2) $$\left[\begin{array}{c} L_{1}^{}\\ L_{2}^{}\\ L_{3}^{} \end{array}\right]=\left[\begin{array}{ccc} \Phi_{lase-fuse} & 0 & 0\\ 0 & \Phi_{cam-fuse} & 0\\ 0 & 0 & \Phi_{temp-fuse} \end{array}\right]•\left[\begin{array}{c} w_{1}•J^{lase}\\ w_{2}•X^{cam}\\ w_{3}•Y^{temp} \end{array}\right]+\left[\begin{array}{c} \Delta_{lase-fuse}\\ \Delta_{cam-fuse}\\ \Delta_{temp-fuse} \end{array}\right]\;$$

where $w_{1}$, $w_{2}$ and $w_{3}$ respectively represent the weights of 3D point clouds, visible images and thermal infrared images in the joint calibration process under the same scene. $\Phi_{lase-fuse}$ is an 3×3 orthogonal matrix representing the rotation relationship between the laser scanning coordinate and the fusion coordinate, and $\Delta_{lase-fuse}$ is a corresponding 3×1 translation matrix. Similarly, $\Phi_{cam-fuse}$, $\Delta_{cam-fuse}$, $\Phi_{temp-fuse}$, $\Delta_{temp-fuse}$ represent the spatial rotation and translation matrix from the visible and thermal infrared camera coordinate to the fusion coordinate. After the correction of the weights of each sensor, different kinds of information in forest environment can be merged in the fusion coordinate through rotation and translation parameters of each sensor are calibrated.

In this paper, the internal calibration of each sensor and the joint external calibration process are accomplished simultaneously with calibration plate placed at different distances and locations. Then, the corresponding linear equations of multiple edges of the calibration plate are selected to model a PNP problem. In the conditional constraint of n≥36, there is a least-squares solution as the initial result for parameters of the multi-sensor joint calibration. In order to eliminate the nonlinear errors caused by human intervention, the Levenberg-Marquardt (LM) method [34] was applied to optimize the joint calibration parameters with the following expression:

(3) $$E=\arg\min\sum_{i\geq 36}a_{i}{\left\|\left[\begin{array}{c} L_{1}^{i}\\ L_{2}^{i}\\ L_{3}^{i} \end{array}\right]-{\left[\begin{array}{c} w_{1}\Phi_{lase-fuse}•J^{lase}+\Delta_{lase-fuse}\\ w_{2}\Phi_{cam-fuse}•X^{cam}+\Delta_{cam-fuse}\\ w_{3}\Phi_{temp-fuse}•Y^{temp}+\Delta_{temp-fuse} \end{array}\right]}^{i}\right\|}^{2}\;$$

where $a_{i}$ represents the weight effect of each edge obtained by the three sensors on the of the objective function E. After the transformation relationship between different sensors are determined, the HFD dominated by 3D point clouds is obtained, in which each point has properties including distance, angle, reflectivity, as well as color and temperature. 

### 4.2. Spot-Divergence Supervoxelization 

As traditional segmentation method based on supervoxels are unsuitable for the complex application of AFMs in face with HFD, of which size is changed due to the occurrence of spot-divergence in complex forestry scenes. Therefore, this paper started with the inherent working principle of laser scanner and proposed the spot-divergence supervoxel representing size-changed character of HFD. In this work, we used 26-neighborhood region constraint to construct mutual topological relationship from the high-dimensional fusion data to supervoxels. Assume that there are *n* HFD in a forestry scene, which form the origin dataset $S:\left\{P_{1},P_{2},\cdots ,P_{n}\right\}$. Then the dataset has been divided into K supervoxels, which constitutes the sets $V:\left\{V_{1},\ldots ,V_{K}\right\}$. The detail of supervoxelization process is divided into the four steps: supervoxel space division, spot-divergence process of HFD, center selection and adjacent partition, and extracting feature vector of supervoxel as following.

#### 4.2.1. Supervoxel Space Division

In the fusion coordinate system, the fusion dataset $S:\left\{P_{1},P_{2},\cdots ,P_{n}\right\}$ with the largest spatial coordinate value in $P_{\max}(x_{\max},\;y_{\max},\;z_{\max})$ and the smallest $P_{\min}(x_{\min},\;y_{\min},\;z_{\min})$ value are selected as the two vertices of the entire cuboid space. The length, height and height of the cuboid space are $L_{x}=\left|x_{\max}-x_{\min}\right|$, $L_{y}=\left|y_{\max}-y_{\min}\right|$, and $L_{z}=\left|z_{\max}-z_{\min}\right|$, respectively. According to actual requirements, we divide all HFD into presupposition spaces of supervoxel with the even length $R_{super}$. Set the supervoxel spaces along the x, y, z direction divided by the number of $n_{x},\;n_{y},\;n_{z}$, we can initially determine the number of preset supervoxels:

(4) $$K=n_{x}\times n_{y}\times n_{z}=\frac{Lx}{R_{super}}\times \frac{Ly}{R_{super}}\times \frac{Lz}{R_{super}}\;$$

The minimum size of supervoxel space must satisfy the constraint conditions:

(5) $$R_{super}>N•R_{voxel}(\max)\;$$

where *N* is the constant coefficient, which is set to 4 in this paper. $R_{voxel}(\max)$ denotes the largest edge length of $P_{n}$ in the fusion dataset, which is proposed on account of the laser beam divergence principle.

#### 4.2.2. Spot-Divergence Process of HFD

**Figure 4:**
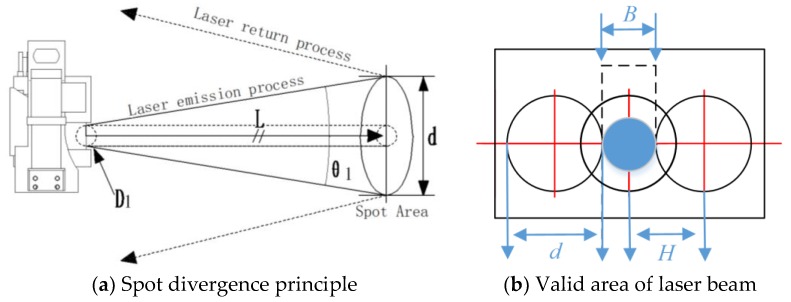
(**a**) Schematic of spot divergence principle with increasing distance. (**b**) The valid area of laser beam is layout of the distance between measured points at different angular resolutions.

The basic starting point is: each laser beam has a divergence angle. As surface reflectivity, texture, roughness, etc. of the object change, the spot-divergence phenomenon of multi-sensor fusion data occurs when the laser beam is reflected back over a long distance. As a result, the spot area of the measured point on the surface of the object is much larger than ever and continuously changes with increasing distance from the laser scanner as shown in Figure 4a. Therefore, describing each HFD with a fixed area size does not meet the actual situation of supervoxels, which leads to inaccurate results of segmentation. Moreover, with increasing distance from the object, the distance between the individual measured points also increases. The distance between the measured points is also dependent on the angular resolution selected. With a coarser resolution (e.g., 0.1667°), the distance is larger, with a finer resolution (e.g., 0.1°) the distance is smaller. To reliably detect an object, the valid area of laser beam with concentrated energy must be fully incident on it once. If the measured laser beam is only partially incident, less energy could be reflected by the object and be disturbed by adjacent beams as shown in see Figure 4b. The size of valid area is proportional to the degree of spot divergence, which represents a lower energy remission than the measured laser beam actually [35]. Therefore, the valid area is applied to describe the size change of each HFD due to spot divergence of 3D point clouds. Based on this idea, a novel supervoxel process was proposed to determine the supervoxel center and adjacent areas. 

The distance-dependent spacing between the measured points is the tangent of the angular resolution × distance. The initial size $D_{1}$ of the laser beam launched from the emitter with the inherent divergence angle $\theta_{1}$ to the surface of the object. After the transmission distance L, the diameter of spot area representing the actual size of each HFD is obtained by the principle of trigonometry as:

(6) $$d=D_{1}+\cot(\frac{\theta_{1}}{2})L\;$$

As shown in Figure 4b, the diameter of each spot area will increase with the distance increases, which leads to overlap of adjacent spot areas. Assume that the center distance of adjacent spot areas is H, which is calculated as:

(7) $$H=2\times L\times \tan(\frac{\varepsilon }{2})\;$$

where ε represents the smallest angle of the adjacent spot areas, which is equal to the pan/tilt angular resolution captured by 3D laser scanner. According to the laser energy distribution, the overlap causes the measurement interference of adjacent laser beam. Therefore, we choose the center area (blue area) as the valid area where there is not mutual overlap and interference of the adjacent beam. The diameter of valid area is defined as:

(8) $$B=H-d/2=2\times L\times \tan(\frac{\varepsilon }{2})-(D_{1}+\cot(\frac{\theta_{1}}{2})L)/2\;$$

**Figure 5:**
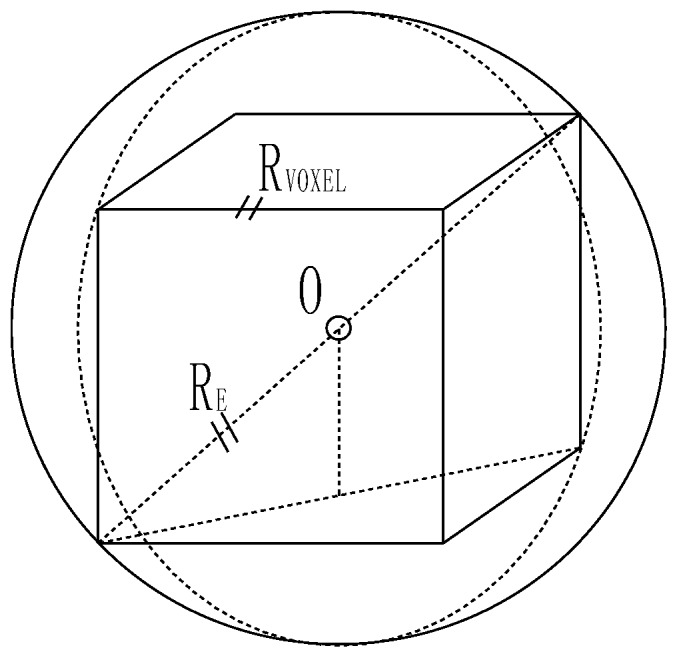
Inscribed cube inside the spheres represents the 3D extension of the valid area of HFD.

According to space division in Section 4.2.1, the cube is the basic computation unit of supervoxel. Therefore, extend the valid area of the laser beam to the 3D space expression, which conform to the realistic geometric distribution of HFD. Then the 2D valid area of each laser beam becomes a 3D sphere with a radius $R_{E}=B/2$. Then, we selected the inscribed cube inside the sphere as the basic element to construct the supervoxel (as shown in Figure 5).

The edge length $R_{voxel}$ of every cube obtained is:

(9) $$R_{voxel}=\frac{2}{\sqrt{3}}R_{E}=\frac{2\times L\times \tan(\frac{\varepsilon }{2})-(D_{1}+\cot(\frac{\theta_{1}}{2})L)/2}{\sqrt{3}}\;$$

Calculate the lengths of all HFD in $S:\left\{P_{1},P_{2},\cdots ,P_{n}\right\}$ and select the maximum value as $R_{voxel}(\max)$. Taking Equation (9) into Equation (5), the size of each HFD and the total number of supervoxels were preset with the practical physical meaning with the spot divergence constraint, which improve the effect of supervoxelization. 

#### 4.2.3. Center Selection and Adjacent Partition 

Based on spot-divergence process, the HFD near the regional center of the supervoxel space is generally selected as the initial seeds. However, in order to avoid the unreasonable situation that the selected data is a noise point or an outlier on the edge position of objects, it is necessary to calculate the size gradient function between the initial seeds and the neighborhood HFD within the search radius Rsearch=Rsuper2 as follows:

(10) $$G(i)=\sum_{k=1}^{N_{seed}}\sum_{j\in N_{adj}}(\frac{\left\|R_{seed}^{i}-R_{voxel}^{j}\right\|}{N_{adj}}+\left\|R_{seed}^{i}-R_{seed}^{k}\right\|)\;$$

where $R_{seed}^{i}$ represents the size of the *i*-th initial seed, $R_{voxel}^{j}$ represents the size value of *j*-th neighboring HFD around this seed. $N_{adj}$ is the number of HFD available in 26-field. $N_{seed}$ represents the number of initial seeds in this supervoxel space, and $R_{seed}^{k}$ represents the *k*-th initial seed within the search range. When G(i) is less than the preset threshold, it indicates that the *i*-th seed meet the constraint requirements and is selected as the central seed of this supervoxel. If the result does not satisfy the constraint, it means that the *i*-th initial seed is invalid. Then the gradient values of different size seeds need to be calculated sequentially until the smallest gradient is selected as the supervoxel center. Subsequently, calculate the spatial distances $d_{adj}^{ij}$ between other HFD and different supervoxel centers for adjacent partition:

(11) $$d_{adj}^{ij}=\sqrt{{\left(x_{i}-x_{j}\right)}^{2}+{\left(y_{i}-y_{j}\right)}^{2}+{\left(z_{i}-z_{j}\right)}^{2}}+\left\|G(i)-G(j)\right\|\;$$

**Figure 6:**
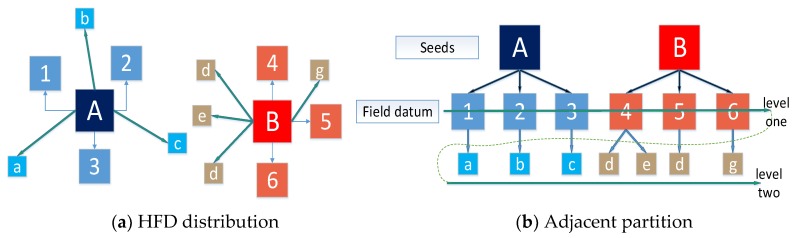
(**a**) Schematic of HFD distribution in two neighbouring supervoxels; (**b**) Schematic of adjacent partition and search process for supervoxels (**b**).

By comparing the distance thresholds $\varepsilon_{1}$, all HFD are allocated to the nearest supervoxel. In order to facilitate the display, this paper uses a schematic diagram to show the partitioning process of two adjacent supervoxels as shown in Figure 6: 

#### 4.2.4. Supervoxel Feature Vector 

With the seed of each supervoxel and neighbouring HFD divided, the large-volume and dense HFD can be divided into small-volume supervoxels distributed sparsely. Each supervoxel can be regarded as a cluster collection of similarity HFD with local characterization, including spatial relations, color, temperature, reflectivity, normal vector and size similarity. Those features are extracted to construct the feature vectors of K supervoxels in high dimensional space as follows:

(12) $$F=\left[{\left(\begin{array}{c} x^{1\sim K}\\ y^{1\sim K}\\ z^{1\sim K} \end{array}\right)}^{T},{\left(\begin{array}{c} L^{1\sim K}\\ a^{1\sim K}\\ b^{1\sim K} \end{array}\right)}^{T},V_{T}^{1\sim K},V_{ref}^{1\sim K},V_{S}^{1\sim K},Var_{S}^{1\sim K},{\left(\begin{array}{c} N_{x}^{1\sim K}\\ N_{y}^{1\sim K}\\ N_{z}^{1\sim K} \end{array}\right)}^{T},T_{h}^{1\sim K}\right]\;$$

The detail properties of each feature mainly include:

1) Spatial coordinates of supervoxel center: $V_{xyz}^{k}=[x^{k},y^{k},z^{k}]$
2) CIELAB color average of *n* HFD in the supervoxel: $V_{Lab}=[\frac{\sum_{i=1}^{n}L_{i}}{n},\frac{\sum_{i=1}^{n}a_{i}}{n},\frac{\sum_{i=1}^{n}b_{i}}{n}]$
3) Temperature average of the supervoxel: $V_{T}=\sum_{i=1}^{n}T_{i}/n$
4) Reflectance average of the supervoxel: $V_{ref}=\sum_{i=1}^{n}r_{i}/n$
5) Edge length $R_{voxel}$ mean of *n* HFD in the supervoxel: $V_{S}=\sum_{i=1}^{n}R_{voxel}^{i}/n$
6) Absolute range between maximum and minimum of $R_{voxel}$: $Var_{S}=\left|R_{voxel}(\max)-R_{voxel}(\min)\right|$
7) Surface normal vector of supervoxel: $N=N_{x}v_{0}+N_{y}v_{1}+N_{z}v_{2}$ with $N_{x}^{2}+N_{y}^{2}+N_{z}^{2}=1$

In this paper, principal component analysis (PCA) is used to calculate the surface normal vector of each supervoxel. The basic principle is calculating the surface normal vector of the approximate plane by minimizing the distance from the surrounding data to the center of supervoxel:

(13) $$d^{2}=\frac{{\sum_{i=1}^{n}\left\|{\left(p_{n}-\bar{p}\right)}^{T}N\right\|}^{2}}{{\left\|N\right\|}^{2}}=\frac{N^{T}•\sum_{i=1}^{n}\left|{\left(p_{i}-\bar{p}\right)}^{T}\cdot \;(p_{i}-\bar{p})\right|•N}{n•{\left\|N\right\|}^{2}}\;$$

where $\bar{p}$ is the local center of supervoxel, and the approximate normal is associated with the smallest eigenvalue $\left(v_{0},v_{1},v_{2}\right)$ of the symmetric positive semi-definite matrix. Searching *n* HFD to determine a local surface normal vector of each supervoxel.

8) Comprehensive dissimilarity of vectors: $T_{h}=\eta_{1}\cdot \arccos\frac{\left|n_{0}•n_{k}\right|}{\left|n_{0}\right|\left|n_{k}\right|}+\eta_{2}\left\|n_{0}-n_{k}\right\|$

where $\theta_{k}=\arccos\frac{\left|n_{0}•n_{k}\right|}{\left|n_{0}\right|\left|n_{k}\right|}$ indicates the angle between the normal vector of *k*-th supervoxel and the Z-axis of the fusion coordinate system. $\left\|n_{0}-n_{k}\right\|$ indicates the numerical vector deviation of *k*-th supervoxel. $\eta_{1}$ and $\eta_{2}$ are weights applied to balance the relationship between angle and deviation of normal vector.

### 4.3. Gaussian Density Peak Clustering 

Compared to traditional clustering methods requiring the artificially preset of clustering central number or convergence thresholds, the density peak clustering (DPC) accomplishes semantic object segmentation adapting to arbitrary shapes and feature types. However, the segment result of DPC excessively depended on the suitable threshold including truncation distance, local density and the minimum higher-density distance, which were all estimated on the basis of empirical experience. This was difficult to segment objects from supervoxels automatically in forestry scenes. Thus, this paper used the normalized feature to construct Gaussian density peak clustering model. With semi-supervised way for extracting threshold, the proposed method can cluster different objects in the forestry environment, which improves accuracy and timeliness of segmentation.

#### 4.3.1. Feature Normalization

As the feature units and quantity levels of supervoxels are very different, each feature channel needs to be normalized by the central regularization process. The Euclidean distance of the supervoxels $F_{k}$ and $F_{q}$ in each feature space is calculated as:

(14) $$\begin{array}{l} d_{1}=\sqrt{{\left(x^{k}-x^{q}\right)}^{2}+{\left(y^{k}-y^{q}\right)}^{2}+{\left(z^{k}-z^{q}\right)}^{2}}\\ d_{2}=\sqrt{{\left(L^{k}-L^{q}\right)}^{2}+{\left(a^{k}-a^{q}\right)}^{2}+{\left(b^{k}-b^{q}\right)}^{2}}\\ d_{3}=\sqrt{{\left(V_{ref}^{k}-V_{ref}^{q}\right)}^{2}+{\left(V_{T}^{k}-V_{T}^{q}\right)}^{2}}\\ d_{4}=\sqrt{{\left(V_{S}^{k}-V_{S}^{q}\right)}^{2}+{\left(Var_{S}^{k}-Var_{S}^{q}\right)}^{2}}\\ d_{5}=\sqrt{{\left(N_{x}^{k}-N_{x}^{q}\right)}^{2}+{\left(N_{y}^{k}-N_{y}^{q}\right)}^{2}+{\left(N_{z}^{k}-N_{z}^{q}\right)}^{2}}\\ d_{6}={\left\|T_{h}^{k}-T_{h}^{q}\right\|}_{2} \end{array}$$

After the weights of the influence of spatial distribution, color difference, temperature and reflection difference, edge length difference, normal vector difference, and synthetic similarity are assigned, all different features are set in the range 0–1, then the high-dimensional distance $D_{kq}$ is obtained:

(15) $$D_{kq}=\left\|\sum_{j=1}^{6}\tau_{j}d_{j}{}^{2}\right\|\;$$

#### 4.3.2. Gaussian Local Density Distribution 

According to the density peak clustering, we define the local density of the *k*-th supervoxel as $\rho_{k}$, which is obtained by the interaction between the high-dimensional distance space $D_{kq}$ and the truncation distance $D_{c}$. The following relationship exists:

(16) $$\rho_{k}=H(D_{kq},D_{c})\;$$

This paper assumes that the local density of all supervoxels conforms to a specific Gaussian distribution:

(17) $$\rho_{k}=\frac{1}{(K-1)D_{c}}\sum_{q=1}^{K-1}\frac{1}{\sqrt{2\pi }}\exp\left\{-\frac{D_{kq}{}^{T}•D_{kq}}{2D_{c}{}^{2}}\right\}\;$$

When the $D_{c}$ is small, the local density distribution of $F_{k}$ shows the prominent form of the middle peak. Only supervoxels that are especially close to $F_{k}$ can play a role, which limits the local density function performance range to a small area. With the increase of $D_{c}$, the distribution of local density function also tends to be flattened, making it possible to influence the local densities of different supervoxels. However, the smooth also inhibits the fact that the contribution degree of $\rho_{k}$ on divergence of supervoxels with different feature association. Therefore, the selection of $D_{c}$ affect the segmentation results and needed to be preset in fixed value [30]. In this paper, a proportional coefficient $t=D_{kq}/D_{c}$ is chosen to select the $D_{c}$ value, which represents the proportion of the neighbors number of each supervoxel in the entire HFD dataset. While taking into account the dimension coefficient w=1, the non-parametric the rule of thumb method was used to determine the $D_{c}$ of Gaussian local density function. In order to meet 98% confidence, the ratio 2.58% is selected as the optimal choice according to the actual requirement of AFMs in forestry environmental. The probability strategy of determining the truncation distance through the proportional coefficient reduces the dependence of the parameter on the specific problem to some extent, and the choice of this ratio is simple and applicable to other problems.

#### 4.3.3. Clustering Supervoxels as Objects

Assume that there are K−k supervoxels with higher local density than the *k*-th supervoxel. Apply the expression (15) to calculate the distance between these supervoxels and $F_{k}$, and form the distance vector:

(18) $$W=[D_{a-k},\cdots ,D_{K-k}]\;$$

Subsequently, the minimum value $W_{\min}$ is selected to calculate the minimum higher-density distance of $F_{k}$:

(19) $$\delta_{k}=\{\begin{array}{c} W_{\min},k<K\\ \max_{\rho_{k}}(D_{kq}),k=K \end{array}\;$$

**Figure 7:**
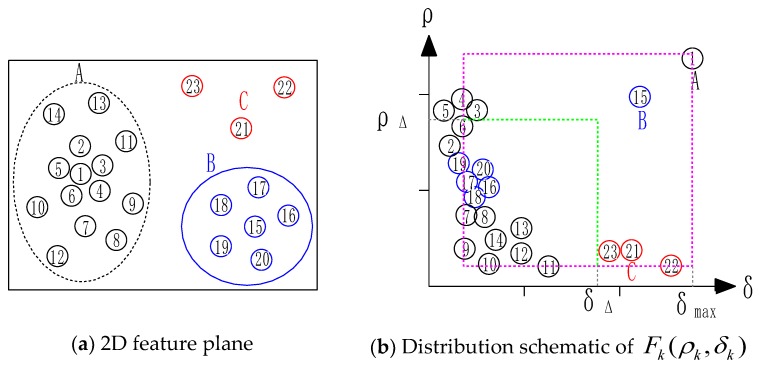
(**a**) Supervoxels projection to 2D feature space data with two clusters; (**b**) The corresponding distribution schematic with $F_{k}(\rho_{k},\delta_{k})$.

If k=K, the super voxel $F_{k}$ is the maximum in the local density ranking, its minimum higher-density distance needs to be redefined. Calculate the higher-density distance from this supervoxel to other supervoxels and select the maximum value as $\delta_{k}$. Then each supervoxel can be expressed as $F_{k}(\rho_{k},\delta_{k})$ with two novel parameters. Draw the distribution schematic of different supervoxels with $\delta_{k}$ as horizontal axis and $\rho_{k}$ as vertical axis: 

As shown in Figure 7 above, there are 22 supervoxels with two actual classifications A and B projection to 2D feature space. In the distribution schematic, the partitioning coefficient $\rho_{\Delta },\delta_{\Delta }$ can be set according to the actual situation to determine the corresponding clustering center. When the $\rho_{k}>\rho_{\Delta },\delta_{k}>\delta_{\Delta }$ constraints are satisfied, the supervoxel can be considered as a clustering center. If there is a case where the minimum distance is large but its density value is less than the threshold, it is defined as an outlier noise point and it needs to be eliminated. By selecting the density threshold and the higher-density distance threshold dynamically, the cluster centers are determined without the number preset of clusters in advance. However, the preferable thresholds $\rho_{\Delta },\delta_{\Delta }$ need to be selected by human observation and intervention. Thus, a novel comprehensive evaluation expression $\gamma_{k}=\delta_{k}•\rho_{k}$ is proposed to select the cluster centers in semi-supervised way as follows:

(20) $$F_{c}=F_{k}(\gamma_{k}>\gamma_{\Delta })\;$$

Through this optimization process, the $\gamma_{k}$ of all supervoxels are calculated and ranked in descending order of density values:

(21) $$\gamma_{K}>\cdots >\gamma_{K-m}>\gamma_{\Delta }>\cdots >\gamma_{1}\;$$

where $\gamma_{\Delta }$ is judgment threshold, which equivalent to finding the number of supervoxels significant improved than other supervoxels. Through this semi-supervised method without manually observe, the number of clusters can be achieved automatically. When m supervoxels are identified as cluster centers, supervoxels close to each center are selected in density ordering and divided into different areas of several objects $C:\left\{C_{1},\ldots ,C_{m}\right\}$. For any supervoxels $F_{w}$ of a non-clustered center, a cluster center with a larger density is sought in the local density arrangement. The higher-dimensional distance between $F_{w}$ and these center $\left\{peak_{1},\ldots ,peak_{m}\right\}$ is calculated, and the cluster center $peak_{w}$ with the smallest distance is selected as its cluster center, which defines $F_{w}$ as the corresponding neighborhood. In order to determine the classification of different super voxels:

(22) $$\left\{peak_{1},\ldots ,peak_{m}|\rho_{center_{m}}>\rho_{F_{w}}\right\}\to D(F_{w},\left\{peak_{1},\ldots ,peak_{m}\right\})=\min\;$$

(23) $$C_{w}:near(F_{1},\ldots ,\;F_{w})\to peak_{w}\;$$

**Figure 8:**
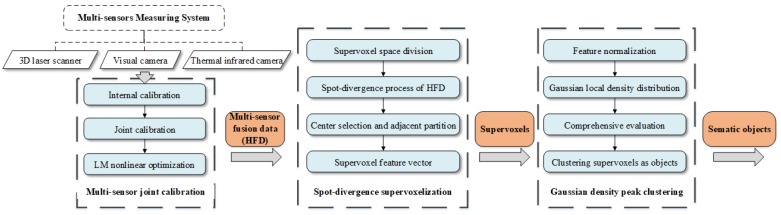
The analysis flow of semi-supervised segmentation framework for AFMs.

Compared with the way that all supervoxels need to traverse the calculation relationship with all center, this method only calculates the relationship between centers and adjacent higher-density super voxels, which can reduce calculated quantity effectively and improve the speed of neighborhood division. Finally, the overall analysis flow of our segmentation framework is shown in Figure 8, and is mainly divided into three consecutive phases as following:

## 5. Results and Analysis

### 5.1. Multi-Sensor Fusion Evaluation

**Figure 9:**
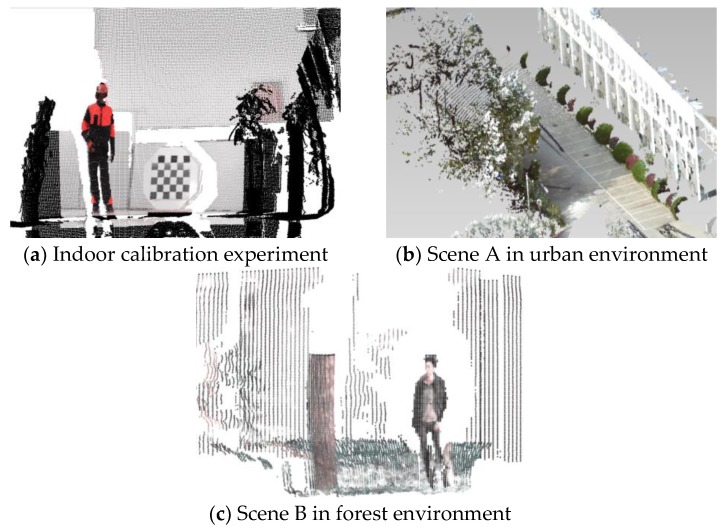
(**a**) Joint calibration experiment was accomplished in indoor scene with well fusion performance; (**b**) HFD of Scene A were obtained in urban environment without extra calibration process; (**c**) HFD were obtained in Scene B of forest environment at extreme distances.

The multi-sensor joint calibration process presented in the previous section was programmed with the octagonal calibration plate. The calibration experiment was accomplished in indoor scene as shown in Figure 9a. By posing the calibration plate in different positions and distances, the internal and mutual relationship of multi-sensor coordinates were confirmed to fuse visible images and thermal infrared images with 3D laser point clouds. On this basis of joint calibration process, the coordinate relationship of the three measuring devices was kept constant, then the multi-sensor measuring system was directly mounted in various AFMs to capture high-quality HFD in urban and forest scenes without repeating calibration. As the result, objects with fused information including 3D space, color, temperature etc. could be displayed on a human-computer interface of the measuring system for AFMs operation.

In order to illustrate the performance of HFD, this study selected partial data of the urban environments in Figure 9b to define Scene A, which was captured in the Jiufeng forest farm during the cold winter season. As a comparison, Scene B was extracted from the forest environments in the artificial eucalyptus farm of Qinzhou during the hot summer and autumn. Both Scene A and Scene B contain six objects such as tree, shrub, pedestrian, stone, building and ground, which were more complicated. In general, Scene A was large-scale displayed in the range of 0.7 m to 40 m with horizontal angle ranging from −5° to 185° and vertical angle ranging from −70° to 70°. Since the measured object was relatively obvious mutual occlusion and measuring temperature is relatively low, the multi-sensor data were well fused in relatively tight form. It’s proved that the measuring system constructed in this paper could cope well with the perception task of urban environment. In order to show the fusing performance of the proposed calibration work in more complex environments, fractional HDF of Scene B were selected to display in Figure 9c at extreme distances ranging 45 m to 50 m (the preset maximum distance of laser scanner). As a result, the HFD were relatively sparsely arranged subject to limitations of laser scanner with spot-divergence. And the visual information was ambiguous during to collective effects of high temperature, low reflectivity of objects and background interference. However, such fusion effect has met the application requirements of AFMs in the forest scenes, and it also proved the necessity of subsequent supervoxels based on spot divergence in this paper.

To objectively evaluate the fusion performance of calibration process optimized by the LM nonlinear method, some analysis results are presented in Table 1. Compared with the calibration method in our previous work [33], the edges relationship of the octagonal plate based on the LM method reduced the calculated value of the average calibration offset error to 2.764 cm and repressed the average angle error to 0.553° effectively with better calibration accuracy, which made HFD suitable for segment application in forest environment. Moreover, the root mean square error (RMSE) in this paper was closed to 5.126, which showed the obvious improvement of nonlinear optimization. Then the corresponding Standard Deviation (STD) was 13.032, which mean that the error distribution was not very discrete and the calibration process was much stable and robust for the following supervoxelization.

### 5.2. Supervoxelization Evaluation 

We conducted experiments on Scene A and Scene B to evaluate the quality of the supervoxels generated by the proposed spot-divergence algorithms. There were 318,331 original HFD in Scene A and 339,547 original HFD in Scene B, which is a relatively large computation for the workload of point-level segmentation. Based on the obtained HFD, the proposed supervoxelization method based on the laser divergence scale change were applied to determine the supervoxel center and search relationship of adjacent areas. With the large-volume and dense HFD divided into small-volume supervoxels sparsely, each supervoxel contains HFD with similar properties of local features. In order to execute and run the supervoxelization method of this article, a workstation machine with 32 GB memory, 500 G SSD and the Intel Core 7 core processor was chosen for model calculation. Then the software platform Point Cloud Library (PCL) for supervoxelization [36], which was an open source programming library run on Ubuntu system.

After the changes from HFD to supervoxels, non-ground 135,369 HFD were converted to 14,240 supervoxels, and other 155,040 HFD on the ground were converted to 7602 supervoxels. The remaining 27,922 HFD were discriminated as noise and deleted in the Scene A. Similarly, the ground of Scene B originally had 200,985 HFD, and after the change, 8357 supervoxels were obtained, while the non-ground was converted from 127,546 HFD to 10,714 supervoxels, and the remaining 11,016 HFD were identified as noise. Obviously, supervoxelization could reduce the amount of computation and improve the efficiency of segmentation.

**Figure 10:**
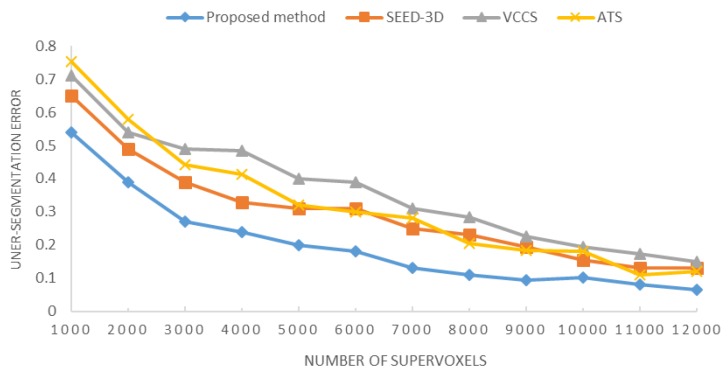
Relationship between under-segmentation error and the number of supervoxels for VCCS (gray), SEED-3D (orange), ATS (yellow) and the proposed method (blue).

To evaluate the performance of our algorithm, it is reasonable to compare the proposed method with algorithms that were also designed to generate supervoxels. We compared our method with three of these kinds of algorithms, including VCCS [21], (vSLIC), SEED-3D [22], and ATS [24], whose source codes were publicly available at their respective research websites. We used the default parameters provided by their authors for all the compared methods. Comparisons of some early methods that segment fusion data without considering the property of spot-divergence could be found in Figure 10. As shown, a further analysis on the ability adhere to object boundaries was developed. Under-segmentation error was chosen as the standard measure for boundary adherence (namely, the error between the given region from the ground truth segmentation and the set of supervoxels required to cover it in minimum number). Then the relationship between the under-segmentation error and the number of supervoxels was shown as following:

Since the number of super voxels increased, the over-segmentation errors of the four methods show a decreasing trend. As plotted above, the blue curve repressing the proposed supervoxelization outperforms the other methods in under-segmentation error, showing the lowest undersegmentation error for most of the useful operating regime. It also means the supervoxel partitioning based on the spot divergence constraints is a better approach, which tightly fitted the ground truth result of object edge in complex scenes. 

**Figure 11:**
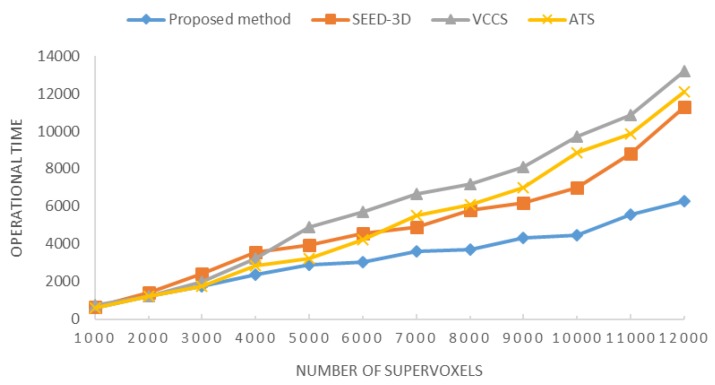
Relationship between the operation time and the supervoxel number.

Further, supervoxels were often proposed to replace the point-wise operation to help speed up segmentation algorithms, which mean that it is important to generate lots of supervoxels efficiently in the first place. Thus, we compared the operational time required for the various methods to segment HFD with the same hardware platform in Figure 11.

With increasing size on number of supervoxels, the operation time curves of all methods increased with the corresponding O(N) complexity. Comparing with curves of VCCS (gray), SEED-3D (orange) and ATS (yellow), the spot-divergence-based algorithm was the fastest supervoxel method, and its advantage increased with the size of supervoxel magnitude. While the operation time of other methods were greatly affected by the increasing trend of supervoxels’ number, especially in the range of 9000 to 12,000. It showed a significant gap in processing speed and memory efficient in order to handle large multi-sensor fusion data, which can not only reduce the redundancy in subsequent data processing, but also facilitate the feature extraction of complex environment.

### 5.3. Semantic Segmentation Evaluation

**Figure 12:**
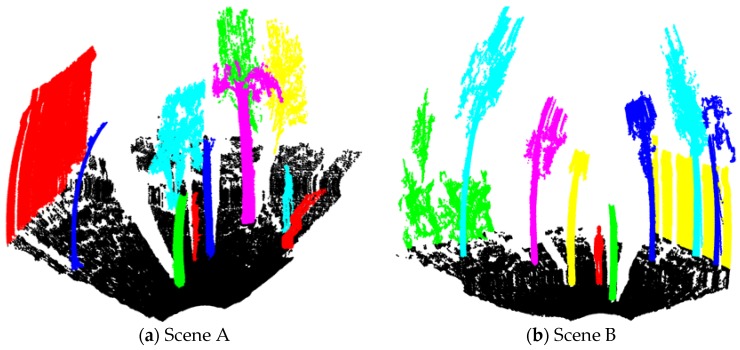
Semantic segmentation of independent objects in Scene A (**a**) and Scene B (**b**).

**Figure 13:**
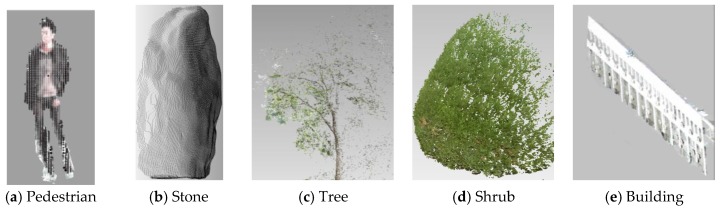
Five types of objects segmented from Scene A and B.

This section tested the semantic segmentation based on density peaks clustering for Scene A and Scene B. as shown in Figure 12. With the supervoxel features. Scene A was segmented semantically as 13 categories, including four trees, three shrubs, one building, two pedestrians, one stone, and two grounds respectively. Scene A was divided into 11 objects, including seven trees, one shrub, one building, one pedestrian, and one ground. Each target is randomly assigned a color to distinguish. Obviously, this algorithm can effectively segment supervoxels as different types of independent objects with small error, as shown in Figure 13.

In order to further evaluate the segmentation performance, the artificial manual segmentation was used as the standard segmentation results, and compared with the segmentation results obtained by the proposed segmentation method. The association matrix of segmentation results for Scene A and Scene B were shown in Table 2 and Table 3. Each element in the table represents the corresponding supervoxels of the actual output label. If a supervoxel is segmented to the correct target, it is called true positive *TP*; if a supervoxel is not segmented but assigned to a nearby target, it is called false negative *FN*; if a target does not exist but a supervoxel is wrongly segmented to it, it is called false positive *FP*. Calculate the precision rate Per, recall rate Rec, and F1-score value F of each scene separately to achieve the evaluation of the segmentation effect:

(24) $$Per=\frac{TP}{TP+FN},\;Rec=\frac{TP}{TP+FP},\;F=\frac{2\times Per\times Rec}{Per+Rec}\;$$

where Per measures the probability between the number of supervoxels correctly segmented for a certain class and the true total number belonging to that class in the artificial standard results. And Rec is the ratio between the number of supervoxels correctly segmented and the total number of supervoxels assigned to the class in this segmentation methods, which describes the probability of objects that can be extracted from supervoxel feature by our method. The *F* value indicates the harmonic mean evaluation of precision and recall. 

Experiments showed that the proposed algorithm achieved very competitive results in the individual objects segmentation in complicated scenes. From the result in Table 2, it was concluded that the accuracy and recall of stones were slightly poorer because supervoxels of stones were partly divided into ground and pedestrian in many cases. However, the comprehensive segmentation of all objects maintained a high value, which validated the performance and stability of the proposed segmentation method. Table 3 showed that Scene B had one less category of stone than Scene A, which improved the evaluation results of segmentation with small amplitude. Moreover, the excellent performance proved that the algorithm retained the characteristics of the original HFD, which were adaptable to both urban and forest environments. Nevertheless, the comparison result also indicated that the semantic categories and individual number of objects affected directly segmentation capability of this proposed method, which were associated with environmental distribution and various attributes of raw datum obtained by the multi-sensor measuring system. The detail results of the proposed algorithm were shown as following.

**Figure 14:**
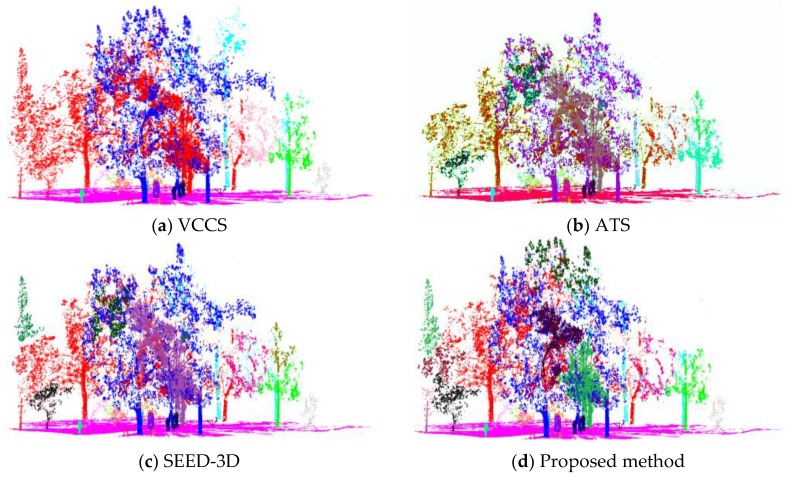
Comparison of segmentation results with the method of VCCS, ATS, SEED-3D in K-means clustering, and the proposed method in Gaussian density peak clustering.

In order to further verify the applicability and robustness of the proposed pipeline, we conducted a comparative trial on the Scene C extracted from forestry environment. The scene was relatively complex manually judged as 43 sematic objects. The proposed method automatically divided Scene C into 44 objects including four big stones, six pedestrians, one ground, 27 trees, two buildings and four shrubs, which was close to the result of manual segmentation with one more tree. Looking in the scene, we found that too many objects were obtained at once, resulting in mutual occlusion and data interference, which was the source of this problem. Wherever, this method still maintained better segmentation performance than comparison methods as shown in Figure 14:

The K-means clustering was combined with supervoxel for sematic segmentation in VCCS, SEED-and ATS to construct the comparison methods respectively. With the preset parameters of 43 clusting centers and 2 m search radius, we could see the segmentation performance among different approaches displayed intuitively. As can be seen, the results of VCCS was presented as 39 final spectral classes different objects, resulting in severe over-segmentation and poor performance even with supervoxels. In Figure 14b, using supervoxel as the neighborhood allowed one to better discern differences inside and between tree areas. Results showed that, despite the ATS method taking only 3D point clouds for supervoxel generation, the subtle differences associated with the main trees and the other objects were properly represented. However, due to the lack of other information in HFD, the method easily divided the discrete data into more object classes or noise clusters in large-scope. With fusing various information of HDF, SEED-3D achieved a better performance of sematic segmentation presented in the complex environment. Although SEED-3D also correctly detected the various objects closing to manual results, this algorithm was easy to assign the same object with different labels in Figure 14c. The main reason was that the features of SEED-3D cannot represent the size-changed character of HFD due to the spot divergence and this process required the presetting parameters of K-mean under human intervention consequently, which needed to be tuned by experiments for achieving the optimal results in different scenes. As a consequence, for both supervoxelization and sematic segmentation, the proposed method better reflected the distribution and features of objects in the HFD, showing a notable variety in a semi-supervised way. In order to compare the overall performance among different approaches in a statistically-rigorous fashion, the statistical significance of differences in terms of accuracy and operation time were evaluated in Table 4 as following:

As the results summarized in Table 4 show, the proposed segmentation framework accounted for an accuracy improvement of the overall sematic segmentation performance in many forest stands. Obviously, the integrated clusters and discrete clusters showed the segmentation work can be performed reasonably according to the environmental characteristic in the semi-supervised case. Another strength of the proposed techniques was that the accuracy and quantity utilization of HFD was significantly improved (see *F* value). This is mainly due to the fact that the spot-divergence supervoxel must be more precise than the stationary supervoxel or point clouds. The performance of supervoxel-based sematic segment depends on the multi-sensor data density and the forest type. Thought the proposed approach achieve an improvement in operation time, the major limitation of our work is that the whole time cannot meet real-time applications of AFMs. Thus, however, the achieved improvement of the overall time would be the focus of subsequent work, which is necessary to the real-time perception of AFMs in forest environment.

## 6. Conclusions

In this paper, we have focused on a semi-supervised segmentation framework based on a spot-divergence supervoxelization of multi-sensor fusion data acquired by AFMs in complex environments. On the basis of multi-sensor measuring system, we have presented a novel three-step segmentation framework representing a semi-supervised processing workflow: Firstly, the relationship of multi-sensor coordinates was joint calibrated to form higher-dimensional fusion data. Secord is given by a spot-divergence supervoxelization instead of producing immutable supervoxels. The novel supervoxel took the size change of each HFD into account to produce feature vectors covering the valid information at a time. Finally, the Gaussian density peak clustering was proposed to segment supervoxels into sematic objects in the semi-supervised way, which non-required the artificially preset of clustering central number or convergence thresholds. Experiments demonstrated that the proposed framework performed well in terms of segmentation accuracy and operation time, which was much appropriate to applications of AFMs. For future research, we would focus on real-time improvement in sematic segmentation of objects. We would also like to extend the method to a more complex scene such as the food security of the grain & oil supply chain.

## Figures and Tables

**Table 1:** Analysis result of multi-sensor joint calibration.

Parameters	Calibration Work in [33]	Proposed Calibration Work
Average calibration offset error	5.819 (cm)	2.764 (cm)
Average angular error	1.164°	0.553°
RMSE	8.232	5.126
STD	19.823	13.032

**Table 2:** Evaluation results of six objects segmentation in Scene A.

	Ground	Pedestrian	Tree	Shrub	Building	Stone	Average
Ground	14048	12	17	63	76	24	
Pedestrian	6	410	5	1	0	11	
Tree	13	2	2208	65	3	5	
Shrub	19	4	35	1526	13	6	
Building	26	0	9	12	2672	15	
Stone	15	16	0	4	9	492	
Precision	0.987	0.947	0.962	0.952	0.977	0.918	0.957
Recall	0.994	0.923	0.971	0.913	0.964	0.890	0.943
F value	0.990	0.935	0.966	0.932	0.970	0.904	0.950

**Table 3:** Evaluation results of five objects segmentation in Scene B.

	Ground	Pedestrian	Tree	Shrub	Building	Average
Ground	8145	24	26	149	13	
Pedestrian	32	710	4	19	1	
Tree	66	7	5341	65	6	
Shrub	19	4	35	3476	1	
Building	44	11	29	12	832	
Precision	0.975	0.927	0.974	0.983	0.897	0.951
Recall	0.981	0.939	0.983	0.934	0.975	0.962
F value	0.978	0.933	0.978	0.958	0.934	0.956

**Table 4:** The segmentation evaluation of four segmentation algorithms.

Segmentation Algorithm	Integrated Clusters	Discrete Clusters	F Value	Time (Approximate)	Effective HDF
VCCS [21] + K-mean	39	317	0.893	92 min	803,252
SEED-3D [22] + K-mean	45	382	0.938	51 min	756,328
ATS [24] + K-mean	48	426	0.920	65 min	983,174
Proposed method	44	125	0.942	34 min	1,139,829

Note: Effective HDF means Number of data for all integrated clusters (larger the value, less data is lost).
